# Role of Neutrophil-to-Lymphocyte Ratio (NLR) in Patients with Mycosis Fungoides

**DOI:** 10.3390/diagnostics13111979

**Published:** 2023-06-05

**Authors:** Cosimo Di Raimondo, Paolo Lombardo, Cristiano Tesei, Fabiana Esposito, Federico Meconi, Roberto Secchi, Flavia Lozzi, Alessandro Monopoli, Maria Grazia Narducci, Enrico Scala, Cecilia Angeloni, Alberto De Stefano, Siavash Rahimi, Luca Bianchi, Maria Cantonetti

**Affiliations:** 1 Department of Dermatology, University of Roma Tor Vergata, 00133 Rome, Italy; flavia.lozzi@hotmail.com (F.L.); luca.bianchi@uniroma2.it (L.B.); 2 Istituto Dermopatico dell’Immacolata, IDI-IRCCS, 00167 Rome, Italy; lombardo.paolo89@gmail.com (P.L.); a.monopoli@idi.it (A.M.); narducci@idi.it (M.G.N.); e.scala@idi.it (E.S.); s.rahimi@idi.it (S.R.); cantonet@uniroma2.it (M.C.); 3 Department of Hematology, University of Roma Tor Vergata, 00133 Rome, Italy; c_tesei@yahoo.it (C.T.); fabiana.e91@gmail.com (F.E.); federico.meconi89@gmail.com (F.M.); robsecchi922@gmail.com (R.S.); 4 Department of Diagnostic Imaging and Interventional Radiology, University of Roma Tor Vergata, 00133 Rome, Italy; cecilia.angeloni@live.it; 5 Volunteers Association of Fondazione Policlinico “Tor Vergata”, 00133 Rome, Italy; alberto.destefano@uniroma2.it

**Keywords:** mycosis fungoides, CTCL, lymphoma, NLR

## Abstract

Background: The neutrophil/lymphocyte ratio (NLR) at baseline has been demonstrated to correlate with higher stages of disease and to be a prognostic factor in numerous cancers. However, its function as a prognostic factor for mycosis fungoides (MF) has not been yet clarified. Objective: Our work aimed to assess the association of the NLR with different stages of MF and to outline whether higher values of this marker are related to a more aggressive MF. Methods: We retrospectively calculated the NLRs in 302 MF patients at the moment of diagnosis. The NLR was obtained using the complete blood count values. Results: The median NLR among patients with early stage disease (low-grade IA-IB-IIA) was 1.88, while the median NLR for patients with high-grade MF (IIB-IIIA-IIIB) was 2.64. Statistical analysis showed positive associations of advanced MF stages with NLRs higher than 2.3. Conclusions: Our analysis demonstrates that the NLR represents a cheap and easily available parameter functioning as a marker for advanced MF. This might guide physicians in recognizing patients with advanced stages of disease requiring a strict follow-up or an early treatment.

## 1. Introduction

Cutaneous T-cell lymphomas (CTCL) represent around 75–80% of all primary cutaneous lymphomas (PCLs). PCLs constitute a variety of non-Hodgkin lymphomas (NHL) with exclusive clinical and histological characteristics, with an incidence rate of approximately 6.4–7.7/1,000,000 person-year in the United States [1]. Contrarily, the prevalence of CTCL is rather elevated due to the excellent survival for the majority of clinical and histological subcategories, with around 25,000 to 50,000 individuals living with CTCL in the United States [2].

### 1.1. Epidemiology

Mycosis fungoides (MF) and Sézary syndrome (SS) represent the most frequent types of cutaneous T-cell lymphoma, with MF being the most frequent subtype and covering almost 60% of all CTCLs. Sézary syndrome, on the other hand, is rarer and encompasses approximately 2.5–3% of cases, showing a probable incidence of 0.3 per million [3]. In Italy, MF is characterized by a very low incidence: the 2015 AIRTUM report on rare tumors documented an incidence of MF of 1.07, with greater frequency in males (1.40) compared to females (0.76) and a greater incidence of patients >65 years of age. Considering the delay in reaching a correct diagnosis due to the well-known clinical and histological overlap with many inflammatory and neoplastic skin diseases, the real impact on society from this rare disease might be higher than expected [4]. The median diagnostic delay in a study on 157 patients was 2.3 years and depended on whether the diagnosis was established after one or multiple biopsies [5]. Mycosis fungoides normally involves elderly patients, aged around 55–60 years with a peak near 80 years, showing a male-to-female ratio that varies from 1.6 to 1.9:1. SS has also been demonstrated to mainly affect men and elderly patients [6].

### 1.2. Pathogenesis

Due to the rarity of this wide spectrum of disease, the precise pathogenesis of CTCL is largely unknown and is probably ascribable to more than one etiology [7]. CTCL takes its origin from clonal proliferation of central memory CD4^+^ T cells in a background of persistent inflammation; that creates a protumorigenic environment that surrounds tumor cells. It has been demonstrated that chronic antigenic stimulation along with cytogenetical aberrations lead to clonal expansion of memory T cells of the skin, which underlies the pathogenesis of CTCL [8]. Another role in the pathogenesis of CTCL seems to be played by bacterial and viral agents [9]. In particular, it has been shown that skin colonization with *Staphylococcus aureus* is linked to disease progression, while eradication of the same pathogen is correlated with clinical remission [10]. Furthermore, it has been assessed that skin microbiota has a key role in altering skin phenotypes in patients with MF [11,12]. Zhang et al. demonstrated that different signatures in the skin microbiota correlate with different clinical manifestations. They investigated the link between changes in the cutaneous microbiota and symptoms intensity, showing that an increase in *Staphylococcus* in the lesions was associated with more evident erythema. Soreness and more infiltrated lesions were correlated with a low concentration in *Propionibacterium* [13]. Lastly, immunosuppression is believed to represent an additional risk factor in the onset of CTCL. In particular, a higher risk has been documented in HIV-positive patients who underwent organ transplantation and in patients treated with anti-TNFα [14,15,16].

### 1.3. Genetics

Despite numerous studies to date, defining the genetic basis of CTCL could still be challenging. Recently, whole exome sequencing (WES), genomic analysis via comparative genomic hybridization (CGH) analysis, whole genome sequencing (WGS), and RNA-sequencing have disclosed many cytogenetic aberrations that could help in the comprehension process of the pathogenesis of MF and SS. Even though the precise implication of these genetic mutations, namely, chromosomal imbalances (CIs), somatic copy number variants (SCNV), somatic single nucleotide variants (SSNV), and epigenetic modifications, is still undetermined, this information may have possible impacts in the therapeutic landscape [17]. Mao et al. performed a CGH analysis in 18 patients with Sézary syndrome and 16 patients with MF and then associated the results with the findings of more conventional cytogenetics, fluorescent in situ hybridization (FISH), and allelotyping studies. They discovered that the most common mutations were losses involving chromosome 1p, 10q/10, and 19, and gains involving 4/4q, 18, and 17q/17, more common in SS than in MF [18]. Another CGH analysis in 32 patients with CTCL depicted CIs in 21 patients (66%). The most demonstrated mutations were losses of chromosomal regions 17p, 13q, 10q, and 6q, and gains of chromatin of chromosome 7, 8q, and 17q11q22. In advanced stage CTCL, the most recurrent aberrations were losses compared to gains. Notably, authors found a direct correlation between the number of chromosomal aberrations and the clinical stage, suggesting that genes encoded on these loci might impact the pathogenesis of lymphoma and disease progression as well [19]. An additional analysis of 88 SS and 40 IV stage MF patients revealed recurrent genomic losses at 9p21.1 (CDKN2A), 10p11.22 (PTEN), and 13q14.2 (RB1). Furthermore, in a recent study, we investigated the miRNA and mRNA expression profile using RNA-seq analysis in lesional skin samples of 28 patients with mycosis fungoides with large-cell transformation (LCT-MF), a feature that is generally associated with poor response and dismal prognosis. We found miR-146a and miR-21 to be significantly upregulated and miR-708 to be the most significantly downregulated miR in LCT-MF. Ingenuity pathway analysis (IPA) demonstrated the involvement of genes for ICOS-ICOSL, PD1-PDL1, NF-κB, E2F transcription, and molecular mechanisms of cancer-signaling pathways. Moreover, our data indicated that miR-146a, -21, and -708 are associated with the immunosuppressive tumor microenvironment in LCT-MF.

### 1.4. Clinical Features

MF, in the early stage, usually appears as an indolent disease with scaly, erythematous and pruritic patches/plaques that are sometimes difficult to diagnose due to the clinical and histological overlap with a wide spectrum of inflammatory skin disease [3]. On the contrary, advanced-stage disease has a more typical presentation and is identified by the development of tumors and/or erythroderma. Clinicopathologic variants of MF are folliculotropic MF (FMF), pagetoid reticulosis, and granulomatous slack skin that were included in the newest international classification of cutaneous lymphomas released by the World Health Organization (WHO) in 2018. The most common clinical variant is the folliculotropic, accounting for around 10% of all cases of MF while pagetoid reticulosis and granulomatous slack skin are particularly rare [20]. FMF involves mainly the head and neck region, often associated with alopecia, with the characteristic folliculotropic infiltrates. Pagetoid reticulosis is microscopically characterized by a neoplastic, psoriasis-like, infiltrate confined to the epidermis. Granulomatous slack skin is characterized by a neoplastic infiltrate localized to the dermis and hypodermis in the form of granulomas that destroy elastic fibers [21].

### 1.5. Prognosis

MF is typically associated with an excellent prognosis in the early stages with an overall survival of approximately 10 to 35 years, but more than 25% of patients might experience progression to a more invasive stage disease, ending in a median survival of fewer than 4 years, with just around 13 months in patients with infiltration of lymph nodes [22]. On the contrary, the advanced disease usually presents with skin nodules, erythroderma, and/or nodal/visceral involvement. On the other hand, SS is a severe erythrodermic variant with leukemic involvement [23]. MF might stay stable for many years or it could evolve to an advanced stage [6]. Currently, no prognostic factors have been identified that could help clinicians in the prompt detection of patients at high risk of a dismal prognosis. Besides the stage and involvement of extracutaneous sites, additional possible prognostic factors, namely, sex, age, high serum lactate dehydrogenase (LDH), augmented β_2_-microglobulin, high levels of eosinophils, total IgE levels, and folliculotropism (FT), have been described in MF [24]. Furthermore, a small percentage (~20–50%) of individuals with MF can experience large-cell transformation (LCT), which is defined as the development of several large neoplastic lymphocytes representing ≥25% of the infiltrate and is characterized by a poor prognosis [25,26].

### 1.6. Treatment

Treatment of CTCL, and in particular of MF, is, more than in other conditions, based on the stage of disease. Since many patients with CTCL usually present with an indolent disease with patch/plaque-stage MF, the treatment strategy is mainly based on either observation with a more frequent follow-up (i.e., “watch and wait”) or topical treatments. On the contrary, patients who progress to an advanced stage of MF/SS usually necessitate a more aggressive therapy, leading to the use of various combinations of topical treatments (topical steroids, retinoids, nitrogen mustard, imiquimod), nb-UVB/PUVA therapy, biologic-response modifiers (HDAC Inhibitors, interferon, methotrexate, bexarotene), systemic chemotherapeutic agents (gemcitabine and polychemotherapy), and monoclonal antibodies (brentuximab vedotin and mogamulizumab) [27].

### 1.7. Neutrophils

Neutrophils represent the preeminent class of circulating cells in the blood, created in the bone marrow, representing the main part of the innate immune system [28]. Besides their function against extracellular pathogens, neutrophils also regulate the immunomodulation through crosstalk with other cells of the innate and adaptive immune system, such as T cells, B cells, NK cells, dendritic cells, and macrophage [29]. Moreover, neutrophils have an important function in host defense to the point that any alteration of either the number or the function of neutrophils due to congenital or acquired conditions can lead to deadly infections [30,31].

Neutrophils might also have a negative function in some autoimmune diseases such as systemic lupus erythematosus and rheumatoid arthritis [32,33]. Neutrophils are crucial immunomodulatory cells whose activities can promote tumor progression through the production of free radicals that increase DNA mutations, suppressing the immune response and favoring tumor angiogenesis [34,35]. Through single cell sequencing, authors confirmed phenotypic heterogeneity and functional plasticity of neutrophils, demonstrating that when placed under stress or following various diseases, these cells can modify their structure, upregulating and downregulating pathways to vary gene expression [36]. Some authors have demonstrated that a subtype of myeloid cells can overturn T-cell function either in murine or human cancer [37]. This type of cells, known as myeloid-derived suppressor cells (MDSC), comprise both neutrophils and immature myeloid cells. The real clinical influence of these cells on the activity and stability of the immune system is hard to evaluate and is still controversial. A worthy substitute for MDSCs in peripheral blood could be represented by the absolute neutrophil count (ANC). Indeed, many authors have showed that neutrophils are linked to myeloid-derived suppressor cells (MDSC), thus representing their mature counterparts [38]. Since they might have a negative effect on cellular immune response, calculation of the ANC to absolute lymphocyte count (ALC), named the neutrophil-to-lymphocyte ratio (NLR), could represent a future prognostic factor. The correlation of the NLR and disease progression has been lately evaluated in many inflammatory diseases such as psoriasis, ulcerative colitis, Behcet’s syndrome, and Crohn’s disease [39,40,41,42,43]. In many hematological diseases, such as multiple myeloma [44] and Hodgkin lymphoma [45], the NLR has also been demonstrated to play a role as an independent prognostic factor.

Romano et al. found the NLR to be a predictor of OS and PFS in multiple myeloma patients in a cohort of 309 patients treated upfront with novel agents [44]. Furthermore, they analyzed the role of the NLR in newly diagnosed Hodgkin lymphoma patients, confirming the role of the NLR as a predictor of PFS independently from the stage of disease at diagnosis [45]. Ho et al. evaluated, in a retrospective analysis, 148 patients with newly diagnosed diffuse large B-cell lymphoma, demonstrating the prognostic value of the NLR in those patients [46].

These findings can be applied also in solid cancers where an elevated NLR is correlated to a dismal prognosis [47,48,49,50]. Recently, some authors have also demonstrated how activated neutrophils can produce the neutrophil extracellular traps (NETs) that are net-like structures composed of DNA-histone complexes and proteins [51]. Besides their main function in the neutrophil innate immune response, NETs are also involved in cancer progression by entrapping and serving as an adhesion substrate for cancer cells, thus promoting metastatic dissemination [52,53].

The role of the NLR as an independent prognostic marker has been previously evaluated in mycosis fungoides by Eren et al. [54] and Cengiz FP et al. [55] with controversial results.

Therefore, our work aimed to assess the association of the NLR with different stages of MF and to outline whether higher values of this marker are related to a more aggressive MF.

## 2. Materials and Methods

This study was conducted in accordance with the Declaration of Helsinki and approved by the Ethical Committee of the Istituto Dermopatico dell’Immacolata (identification number 4/CE/2015).

We retrospectively analyzed patients with MF at baseline treated at Policlinico Tor Vergata and the Istituto Dermopatico dell’Immacolata between 2000 and 2013. Eligible individuals were at least 18 years of age and had histologically confirmed, by two board-certified dermatopathologists, diagnosis of MF. The diagnoses were made according to the revised 2018 World Health Organization–European Organisation for Research and Treatment of Cancer (WHO–EORTC) classification and ordered according to the revised staging system for MF/SS based on the tumor–node–metastasis–blood (TNMB) classification system [3]. Significant demographic and clinical information such as age, sex, evaluation of skin tumor burden by the modified severity-weighted assessment tool, MF/SS subtype, and vital status at last contact were gathered from the electronic medical record.

After retrospective selection, we collected patients’ clinical data, namely, age, gender, T-stage, N-stage, and M-stage, from our patient database. In all subjects, complete blood count (CBC) and routine biochemical exams, in order to evaluate liver and kidney functional capacity, were registered before any cutaneous biopsy; the NLR was obtained using data calculated from the CBC count. We excluded subjects with evidence of any active bacterial infection.

### Statistical Analysis

Continuous variables are expressed as the median ± SD; categorical variables are expressed as percentages. Variances amid groups were assessed in univariate analysis using non-parametric tests (the chi-squared test in the case of categorical variables and the Mann–Whitney test in the case of continuous variables). The optimal cut-off point was decided by using ROC curve analysis. A value of *p* < 0.05 was considered statistically significant. All the analyses were performed through RStudio 2021.09.0+351 (RStudio Team (2020). RStudio: Integrated Development for R. RStudio, PBC, Boston, MA, http://www.rstudio.com/ (accessed on 1 October 2022) with the packages “Hmisc”, “psych”, “dplyr”, “ROCR”, and “caTools”.

## 3. Results

Three hundred two patients (208 males and 94 females) with a mean age of 58 years were evaluated. Patients’ characteristics at baseline are shown in Table 1.

Among all patients, 112 (37.1%) were stage IA, 109 (36%) were stage IB, 15 (5%) were stage IIA, 31 (10.3%) were stage IIB, 26 (8.6%) were stage IIIA, and 9 (3%) patients were stage IIIB.

According to criteria proposed by the WHO-EORTC, 236 subjects had low-grade (IA-IB-IIA) MF, whereas 66 patients had high-grade (IIB-IIIA-IIIB) disease [3].

Among the 302 patients, 17 had the histologic diagnosis of folliculotropic MF. Median absolute neutrophils count (ANC) and absolute lymphocytes count (ALC) among all patients were 3.8/mmc and 1.8/mmc, respectively, leading to a median NLR of 2.1.

**Figure 1:**
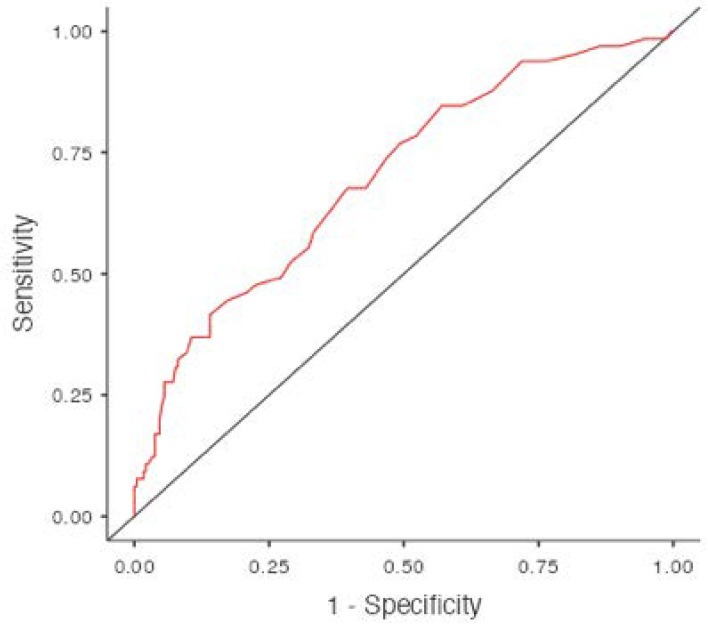
Cut-Off Plot.

The optimal cut-off value of the NLR calculated using ROC curve analysis was 2.3 (67.6% sensitivity and 60.4% specificity), while the AUC (area under curve) was 0.700 (Figure 1).

The median NLR among patients with low-grade IA-IB-IIA MF was 1.88, while the median NLR for patients with high-grade IIB-IIIA-IIIB MF was 2.64 (Table 2).

Progression in stage was recorded in 59 (19.5%) patients. Among them, 28 (16.2%) had NLRs < 2.3 while 31 (24.4%) had NLRs > 2.3 (Table 3).

## 4. Discussion

A high NLR has been demonstrated to have a negative prognostic role in many solid and hematological tumors [56,57,58].

Our study demonstrated the association between high NLR and high-risk/aggressive MF. Thus, those MF patients with elevated NLRs might be routinely targeted for increased clinical and radiological follow-up. In our cohort, statistical analysis showed positive associations among advanced MF stages and NLRs higher than 2.3.

Furthermore, although data regarding progression in stage did not reach statistical significance, we observed that disease progression was higher (24.4%) in patients with NLRs > 2.3 than patients with NLRs < 2.3 (16.2%).

### 4.1. Prognostic Factors

Prognostic factors in MF are more undefined compared to other neoplastic diseases due to the rarity of the disease and diagnostic challenges.

The Cutaneous Lymphoma International Consortium showed the findings of their recently founded Prospective Cutaneous Lymphoma International Prognostic Index (PROCLIPI) international registry for early stage MF [59]. In this project, authors studied around 1000 patients’ data from 29 countries worldwide in order to evaluate clinical, hematological, and radiological data. To date, the clearest prognostic factor in MF patients is the stage of the disease. The stage and the involvement of extracutaneous sites are the most important elements related to survival [60]. Furthermore, a univariate retrospective evaluation showed that advanced age, male gender, increased levels of lactate dehydrogenase, and large-cell transformation are correlated with diminished survival and increased risk of disease progression [61]. A recent analysis showed sex-related differences in MF with a five-year overall survival rate of 76.9% in women versus 70.7% in men, depicting a possible estrogen effect on the proliferation of mycosis fungoides cells [62]. On the contrary, factors related to a better prognosis and a low risk of disease progression are hypopigmented, poikiloderma MF or MF associated with lymphomatoid papulosis (a variant of CD30+ lymphoproliferative disorders that usually have an indolent course) [63,64]. In a previous study, we described the expression profiles of PD1, PD-L1, and ICOS in MF/SS and demonstrated the correlation between elevated expression of these markers and advanced stages of MF, large-cell transformation (LCT), and poor overall survival. Furthermore, we confirmed that PD-L1 is not presented by CTCL cells but is confined on histiocytes/macrophages in the tumor microenvironment, highlighting the important role of the complicated interactions between the neoplastic lymphocytes and various immunoregulators in the tumor microenvironment. Furthermore, it demonstrates that a combined checkpoint marker score might help clinicians in predicting CTCL outcomes and prognosis [23]. Other independent factors predictive of a dismal prognosis are represented by clonality in the absence of Sézary cells and folliculotropic MF, as shown in a recent multivariate analysis, although some authors refer to folliculotropic as a subtype of mycosis fungoides with a favorable prognosis [63]. To date, large-cell transformation remains the most recognized negative prognostic factor. As mentioned above, large-cell transformation consists in the presence of at least 25% of neoplastic lymphocytes four times larger than normal lymphocytes. In a previous study, we studied the miR and mRNA expression profile in lesional skin biopsies of patients with LCT-MF and non-LCT MF using RNA-seq analysis. We demonstrated that miR-146a and miR-21 are considerably upregulated and that miR-708 is the most meaningfully downregulated miR in LCT-MF. We also integrated miR and mRNA expression profiles, revealing the miR-regulated networks in LCT-MF. Ingenuity pathway analysis (IPA) established the contribution of genes for ICOS-ICOSL, PD1-PDL1, NF-κB, E2F transcription, and molecular mechanisms of cancer-signaling pathways. Quantitative real-time (qRT)-PCR results of target genes were coherent with the RNA-seq data. Moreover, we have identified the immunosuppressive tumor microenvironment (TME) in LCT-MF. Therefore, our data revealed that miR-146a, -21, and -708 are associated with the immunosuppressive TME in LCT-MF. Together, our findings suggest that the main LCT-MF-related miRs and their controlled networks may provide new information into its pathogenesis and help find favorable new targets for new treatments [65]. Around 50% of patients with large-cell transformation have the expression of CD30 that can become the target of brentuximab vedotin (BV), an anti-CD30 antibody-drug conjugate recently approved to treat CD30+ cutaneous lymphomas. Its approval followed the ALCANZA study, a phase III trial, that confirmed the safety and the superior efficacy of brentuximab compared to the physician’s choice of methotrexate or bexarotene in refractory pcALCL and MF. Among the 128 patients included in the study as an intention-to-treat population, 97 patients had a diagnosis of mycosis fungoides. Patients randomly assigned to the arm with brentuximab received intravenous brentuximab vedotin 1.8 mg/kg once every 3 weeks for up to 16 3-week cycles, while in the control arm, patients were treated with methotrexate (5–50 mg) or bexarotene 300 mg/m^2^. The final analysis, at a median follow-up of 45.9 months (95% CI, 41.0–49.4), showed a meaningfully improved response, with clinical and symptomatic benefit, to brentuximab vedotin compared to the physician’s choice [66].

### 4.2. NLR in Cancer

An elevated NLR has been widely demonstrated to have a negative role in the prognosis of various solid tumors and hematological malignancies [45,47]. The neutrophil-to-lymphocyte ratio as a prognostic indicator in visceral malignancies has been analyzed in previous reports, underlining an association between inflammation and tumorigenesis that nowadays is commonly recognized [48]. Many authors have implied that an elevated neutrophil-to-lymphocyte ratio (NLR) has a prognostic meaning for inferior outcomes in patients with cutaneous malignancies, such as melanoma, cutaneous squamous cell carcinoma, and Merkel cell carcinoma [50,67,68]. In a retrospective analysis of 97 consecutive patients with metastatic melanoma who received treatment with the anti PD-1 nivolumab, those patients who had NLRs ≥ 5 at diagnosis had considerably lower overall survival and progression-free survival compared to patients with NLRs < 5, demonstrating how the NLR was associated with improved survival when baseline levels were lower than the cut-off values [67]. Zhan et al. performed a meta-analysis on 4593 persons with melanoma, showing that those with elevated NLRs had a considerably lower overall survival and disease-free survival/progression-free survival, suggesting that an elevated baseline NLR was associated with a reduced prognosis in melanoma patients [69]. Among the non-melanoma skin cancers, a retrospective analysis of newly diagnosed cutaneous squamous cell carcinoma patients showed that the median NLR among patients with early stage disease (in situ and stage I) was 2.2, while the median NLR for patients with advanced disease was 4.87. These data demonstrate how high NLRs correlate with higher stages of disease, requiring a strict follow-up and an early treatment for those patients with cutaneous squamous cell carcinoma and a high NLR [50]. Furthermore, in a single-center retrospective case series, Maeda et al., in a previous analysis, analyzed the relationship between the NLR and survival in individuals with squamous cell carcinoma of the skin; furthermore, they studied the correlation between the NLR and sentinel lymph node positivity. Thus, they demonstrated that an elevated NLR correlated with both higher mortality and SLN positivity [70]. Lastly, a similar evaluation was realized by Zaragoza et al., who retrospectively analyzed the role of the NLR in 75 patients affected by Merkel cell carcinoma (MCC). They demonstrated that a value of NLR ≥ 4 among the 75 patients with MCC, and a high NLR at baseline, was associated with a higher death rate but not with recurrence, confirming once again the poor prognosis of a high NLR at baseline [68].

Furthermore, it has been broadly demonstrated that an inflammatory microenvironment is a main factor for all tumors, including those in which the direct link between inflammation and cancer has not yet been fully demonstrated [71]. Therefore, many inflammatory indicators have been evaluated to define their prospective prognostic roles in various malignancies.

### 4.3. NLR in Mycosis Fungoides

To date, only two works have analyzed the role of the NLR as a prognostic index in MF.

Eren et al. were the first authors to explore the neutrophil/lymphocyte ratio and clinical characteristics in a retrospective study of 117 patients with MF. The majority of patients enrolled in the study had an early stage disease that may have influenced the results since it has been widely demonstrated that tumor microenvironment differs remarkably in the early and advanced stages in MF, where the tumor microenvironment in the advanced stages have much more protumorigenic and immune-exhausted expression. In fact, they evaluated whether NLRs ≥ 2 correlated with treatment demand, time to treatment, progression in stage, and time to progression in stage. The authors did not find any association between the NLR and treatment demand, time to treatment, progression in stage, and time to progression, thus demonstrating that NLR had no prognostic relevance in patients with MF [54].

On the other hand, Cengiz FP et al. evaluated the prognostic meaning of the neutrophil-to-lymphocyte ratio in 119 patients with MF. They further evaluated the correlation of the NLR with other factors; namely, age, lactate dehydrogenase, beta-2-microglobulin, and IgE. They found a strong correlation of the NLR at diagnosis in patients who underwent disease progression and those who had a high stage at diagnosis. Furthermore, they observed elevated NLRs in patients with high beta-2-microglobulin concentration, suggesting the function of the NLR in the identification of those patients with high risk of progression. They have reported another strong correlation between the NLR and the lymphocyte/monocyte ratio, which is another marker that has been demonstrated to have a prognostic role in many cancers. Therefore, authors suggest that the NLR can be used to determine a prognosis in MF with beta-2-microglobulin. It may also be useful to make a certain diagnosis of early stages of MF; that, as reported by many authors and, as we see in clinical practice, usually are difficult to diagnose. Compared to beta-2-microglobulin, the NLR would be easier for detection, as it is a potential new prognostic marker for patients with MF [55].

The conflicting results on mycosis fungoides are probably due to different study populations, since the majority of patients in the study by Eren et al. were in the early stages. Our study confirms the association of elevated NLR levels with advanced-stage disease, considering that our study population had higher numbers of advanced-stage patients compared with the two previous studies reported in the literature.

All these data from different studies on many types of cancers confirm the immunosuppressive activity of neutrophils on innate as well as adaptive immunity that contribute to tumor genesis and progression [34].

On the other hand, another key role in the immune response against cancer cells is played by lymphocytes, therefore exhibiting a detrimental impact on disease advancement when their activity is decreased. Lymphocytes also regulate cell proliferation and angiogenesis through the production of antitumoral cytokines [72].

## 5. Conclusions

Common reasoning of many authors is that the NLR can be influenced by many conditions such as patients’ age, body mass index, and alcoholic fatty liver. Indeed, in our cohort of patients, a correlation between the NLR and advanced age was found (*p* 0.005) (Table 2). Our results confirm that the NLR could represent a cheap and easily accessible measure that may be simply acquired from automatic blood cell counts at baseline. Hence, our study highlighted that the NLR may be helpful to evaluate the course of the disease in order to identify patients with a greater risk of aggressive evolution and recurrences. Thus, the NLR can represent an important parameter to be considered in order to select a subgroup of patients that could benefit from a stricter follow-up or a different treatment plan. Some limits of our work are that our study is a retrospective analysis with a small sample of patients. Increasing the number of patients, performing additional prospective analyses, and calculating correlations with progression-free survival and overall survival are required to clearly define the prognostic role of neutrophil-to-lymphocyte ratio in mycosis fungoides.

## Figures and Tables

**Table 1:** Baseline demographic and clinical characteristics of MF patients analyzed in the study.

Characteristics (*n*. of Patients 302)		
Male: Female ratio	(208:94) 2.2	
Median Age, years (range)	58 (18–96)	
Stage	N	%
IA	112	37.1
IB	109	36.0
IIA	15	5.0
IIB	31	10.3
IIIA	26	8.6
IIIB	9	3.0
Grading		
Low (IA-IB-IIA)	236	78.1
High (IIB-IIIA-IIIB)	66	21.9
Progression in stage		
Yes	59	19.5
No	243	80.5

**Table 2:** NLR values according to stage.

	Median ± SD	IQR	*p*-Value
**N/L Low (IA-IB-IIA)**	1.88 ± 1.40	1.325	<0.001
**N/L High (IIB-IIIA-IIIB)**	2.64 ± 7.98	2.520	

**Table 3:** NLR values (<2.3 and >2.3) according to MF staging.

	N/L < 2.3 (*n* = 173)		N/L > 2.3 (*n* = 127)		*p*-Value
Characteristics	N.	%	N.	%	
Male: Female ratio	(117:56) 2.1		(89:38) 2.3		0.651
Median Age, years (range)	55 (18–96)		61 (24–97)		0.005
Stage					
IA	75	45.3	36	28.3	0.002
IB	64	37.0	45	35.4	
IIA	8	4.6	7	5.5	
IIB	11	6.4	20	15.8	
IIIA	14	8.1	11	8.7	
IIIB	1	0.6	8	6.3	
Grading					
Low (IA-IB-IIA)	147	85.0	88	69.3	0.001
High (IIB-IIIA-IIIB)	26	15.0	39	30.7	
Progression in stage					
Yes	28	16.2	31	24.4	0.077
No	145	83.8	96	75.6	

## Data Availability

Data available on request due to restrictions eg privacy or ethical.
